# Risk of Multidrug Resistant Bacteria Acquisition in Patients with Declared *β*-Lactam Allergy during Hospitalization in Intensive Care Unit: A Retrospective Cohort Study (2007-2018)

**DOI:** 10.1155/2022/8906316

**Published:** 2022-01-12

**Authors:** Alessio Strazzulla, Maria Concetta Postorino, Nabil Belfeki, Laura Iordache, Astrid de Pontfarcy, Aurelia Pitsch, Pierre Leroy, Sebastien Jochmans, Mehran Monchi, Sylvain Diamantis

**Affiliations:** ^1^Internal Medicine Unit, Groupe Hospitalier Sud Ile de France, Melun, France; ^2^Public Health Unit, Groupe Hospitalier Sud Ile de France, Melun, France; ^3^Internal Medicine Unit, Centre Hospitalier Sud Seine et Marne, Fontainebleau, France; ^4^Infectious Diseases Unit, Groupe Hospitalier Sud Ile de France, Melun, France; ^5^Biology Laboratory, Groupe Hospitalier Sud Ile de France, Melun, France; ^6^Intensive Care Unit, Groupe Hospitalier Sud Ile de France, Melun, France; ^7^Research Unit “DYNAMIC”, Université Paris-Est Créteil, Créteil, France

## Abstract

**Introduction:**

The risk of extended spectrum *β*-lactamase (ESBL) bacterial acquisition in patients with *β*-lactam allergy has been poorly investigated. In a previous study conducted over a 6-year long period (2007-2012), we found that patients with declared *β*-lactam allergy had a higher risk of ESBL bacterial carriage at admission in intensive care unit (ICU), but they had not a higher risk of ESBL bacterial acquisition. We present the final results of the study which was eventually conducted over a 12-year long period (2007-2018).

**Materials and Methods:**

The study included all patients admitted in ICU and receiving antibiotic treatment from January 2007 to December 2018. ESBL bacterial acquisition was the main clinical outcome. Mortality in ICU, multidrug resistant bacterial carriage at admission and discharge were the secondary outcomes.

**Results:**

Overall, 3332 patients were included, 132/3332 (3.9%) were labelled *β*-lactam allergic, while 3200/3332 (96.1%) did not presented *β*-lactam allergy. No significant difference in rates of ESBL acquisition was detected (4/132, 3% vs. 78/3200, 2.4%; *p* = 0.17). Patients with *β*-lactam allergy had higher rates of ESBL bacterial carriage at admission (19/132, 14.4% vs. 248/3200, 7.8%, *p* = 0.01) and at discharge (22/132, 16.7% vs. 351/3200, 11%, *p* = 0.04) than nonallergic patients. No differences in mortality, duration of hospitalization, and carriage of methicillin resistant *Staphylococcus aureus* were reported. Female gender was the only factor associated with *β*-lactam allergy at the multivariate analysis.

**Conclusions:**

This study confirms that patients with declared *β*-lactam allergy had not a higher risk of ESBL bacterial acquisition during hospitalization in ICU. However, they had a higher ESBL bacterial carriage at admission.

## 1. Introduction

*β*-lactam allergy is largely reported in hospital patients [1], even though the documentation is incomplete in 66% up to 84% of cases with lack of allergen identification and description of the reaction [2]. Because of an often unjustified fear of severe drug reactions, physicians have tendency to prescribe antibiotics other than *β*-lactams in these patients [3]. Among the alternative molecules, vancomycine, clindamycine, and fluoroquinolones are the most frequently prescribed [4–6]. As a consequence, increased risk of methicillin resistant *Staphylococcus aureus* (MRSA) carriage and *Clostridioides difficile* disease was reported [7, 8]. Also, an increased risk of infections by extended spectrum beta lactamase (ESBL) bacteria could be expected [9].

In a previous article, we presented the preliminary data of a retrospective study which investigated the characteristics of patients admitted to intensive care unit (ICU) and the risk of multidrug resistant (MDR) bacterial acquisition. During a 6-year long period (2007-2012), we found that patients with declared *β*-lactam allergy had a higher risk of ESBL bacterial colonization at admission than patients without declared allergy. However, the simple size of the “*β*-lactam allergy” group was not very representative (*n* = 45) when compared with the “non *β*-lactam allergy” group (*n* = 1129), reducing the statistical value of the study [10].

In this article, we present the complete results of the study which was conducted over a 12-year long period (2007-2018) before the coronavirus infectious disease-2019 (COVID-19) pandemic.

## 2. Materials and Methods

A retrospective cohort study was conducted in a 350 acute care-bed hospital in the *Ile de France* region in France over a 12-year long period from January the 1st, 2007, to December 31st, 2018. All adult patients admitted in ICU and receiving antibiotic therapy were included. Exclusion criteria included (i) age at admission < 18 years old, (ii) absence of antibiotic treatment during hospitalization in ICU, and (iii) repetitive hospitalization after a first encounter during the study period.

The study was conducted in accordance with Declaration of Helsinki and national and institutional standards. The local institutional review board waived patients' consent obligation due to the retrospective character of the study, according to the French law. Similarly, a written consent form was not proposed to patients because the noninterventional nature of the study, according to the French law [11, 12].

Software used in daily clinical practice (Sillage v17 and CGM Lab channel 1.20.33686) was employed for clinical data collection (patients' history and characteristics). *β*-lactam allergy was defined by the presence in past medical history of a supposed or documented reaction of any grade (low, mild, or life threatening) to at least a *β*-lactam molecule among penicillins, cephalosporins, and carbapenems. The following patients' data were collected: age, gender, *β*-lactam allergy report, simplified acute physiology score II (SAPS-II), shock, mechanical ventilation, and central venous catheter (CVC). Shock was defined as the necessity of vasopressors for maintaining a mean arterial pressure ≥ 65 mmHg [13].

The main clinical outcome was the acquisition of ESBL producer gram negative bacteria during hospitalization in ICU. Secondary outcomes were (i) acquisition of MRSA during hospitalization in ICU, (ii) mortality in ICU, and (iii) duration of hospitalization in ICU. For the definitions of MRSA and ESBL producers' acquisition, two kinds of samples were considered: (i) screening swabs (nasopharyngeal and rectal) were routinely obtained at admission and discharge and (ii) other samples obtained during the hospitalization according with patient's clinical evolution.

Two groups of patients were compared: (i) allergy group (patients reporting *β*-lactam allergy in their medical history) and (ii) no allergy group (patients reporting *β*-lactam allergy in their medical history).

Fisher's exact test (qualitative variables) and Student's *t*-test (quantitative variables) were applied for the univariate analysis. Quantitative variables were presented in the text as mean values. Differences in clinical outcomes and clinical characteristics of patients included in the two groups were compared (allergy group vs. no allergy group). A multiple logistic regression analysis was performed to explore whether or not *β*-lactam allergy was correlated with some patients' characteristics at the admission. Parameters included in multivariate analysis were chosen according to univariate analysis results (*p* ≤ 0.150). Analyses were performed using R, the language for statistical computing (Vienna, Austria, https://www.r-project.org/). Statistical significance was set at *p* < 0.050.

## 3. Results

Overall, 3616 patients' files were identified; 284/3616 (7.8%) files were excluded according to exclusion criteria. Among the 3332/3616 (92.2%) patients included in the study, 132/3332 (3.9%) were labelled *β*-lactam allergic while 3200/3332 (96.1%) did not presented *β*-lactam allergy.

Table 1 shows the characteristics of the population. The allergy group and no allergy group did not differ in biological and clinical characteristics except for the percentage of women (63% vs. 39%, *p* < 0.01).

No significant difference in rates of ESBL acquisition was detected (4/132, 3% vs. 78/3200, 2.4%; *p* = 0.17). Patients with *β*-lactam allergy had higher rates of ESBL bacterial carriage at admission (19/132, 14.4% vs. 248/3200, 7.8%, *p* = 0.01) and at discharge (22/132, 16.7% vs. 351/3200, 11%, *p* = 0.04) than nonallergic patients. No differences in mortality, duration of hospitalization, and carriage of MRSA were reported.

At the multivariate analysis, *β*-lactam allergy was positively associated with female gender (RR =0.4, *p* < 0.01) as shown in Table 2.

## 4. Discussion

This study confirmed the results of the previous one [10]. Patients with declared *β*-lactam allergy had not an increased risk of ESBL acquisition during hospitalization in ICU, but they presented a higher rate of ESBL colonization at admission than nonallergic patients. However, higher rates of ESBL colonization did not impact mortality rates and length of staying.

The most important result of the study was the confirmation that patients labelled *β*-lactam allergic were at risk of ESBL colonization at admission in ICU. It is necessary to localise the study in the French reality to comprehend this result. Indeed, an over prescription of antibiotics has been observed in France since early 2000s [14–16]. This fact stimulated French governmental authorities to conduct three national campaigns for the preservation of antibiotic efficacy from 2002 to 2016. These campaigns achieved a 25% reduction of antibiotic prescriptions in the entire French territory [17, 18]. Notwithstanding, an increase in rates of *Escherichia coli* resistant to cephalosporines was observed in the same period (from 1.3% to 4.2% in community isolates and from 2% to 11.2% in hospital isolates) [19]. In our study, we observed an overall rate of 8.6% ESBL carriage at admission. This result confirms that the risk of carriage of ESBL bacteria is not negligible in French community. This study suggests that antibiotic stewardship programs should be implemented in community, as already proposed by other authors [20].

This study failed in demonstrating an increased risk of ESBL acquisition during hospitalization in ICU. To explain this result, it is possible to evoke different causes. At first, third generation cephalosporins are currently prescribed in patients declaring a penicillin allergy during their staying in our ICU. This strategy is in line with current data about risk of cross-reactivity between penicillin and cephalosporin allergy (1%), and it makes possible to spare other “pollutant” molecules, such as fluoroquinolones [21, 22]. Secondly, restriction antibiotic policies are ongoing in the ICU of our hospital with the purpose of reducing antibiotic consumption and increase the use of alternative molecules to *β*-lactams and the final objective of limit ESBL spread. For example, a decrease in consumption of fluoroquinolones (-85%), carbapenems (-58%), and glycopeptides (-66%) was observed from 2007 to 2014 [23]. Also, de-escalation from broad spectrum molecules to targeted molecules is routinely practiced [24–26]. Third, ICU patients are always isolated in single bed rooms, and preventive measures are usually strictly respected by health personnel, such as contact prevention, hand hygiene, and environmental decontamination. These procedures could have limited the patient-to-patient spread of ESBL bacteria [27, 28].

Except for ESBL carriage at admission, no difference in other clinical outcomes was observed. Notably, *β*-lactam allergy was not associated with mortality and length of staying in ICU. These results are in line with mortality rates and length of staying reported by other authors [29]. They can only partially be explained by the fact that patients labelled *β*-lactam allergic in our study presented a colonization by ESBL producer bacteria rather than infection [28]. Consequently, the impact of ESBL bacteria on mortality and length of staying was scarce. However, our study was limited to ICU staying, and consequently, we could not infer about impact of ESBL colonization on total duration of hospitalization and mortality in other units.

As the previous one, this study confirmed the low percentage of patients declaring *β*-lactam allergy (3.9%) [10]. This is in line with the study by Leone et al. who presented a 5% rate of patients labelled *β*-lactam allergic in their cohort of ICU patients [29]. These percentages are lower than data usually reported in literature. This discrepancy likely reflects an underreporting of *β*-lactam allergy in ICU due to the severity of patients admitted in ICU and the impossibility to collect a full medical history.

Many research questions are proposed by this study. First, the increased risk of ESBL carriage among patients labelled *β*-lactam allergic needs to be confirmed in larger epidemiologic studies in general populations. Then, the factors influencing ESBL acquisition in community should be investigated. Secondly, trend in antibiotic prescription in general practitioner cabinets and long-term care facilities should be investigated. Thirdly, the absence of ESBL acquisition during ICU staying needs to be confirmed by other studies. Finally, differences in antibiotic prescriptions among patients with or without declared *β*-lactam allergy during ICU staying need to be investigated.

## 5. Conclusions

Patients declaring *β*-lactam allergy have not a higher risk of MDR acquisition or death during hospitalization in ICU, but they are at risk of ESBL colonization at admission. The increased ESBL colonization is likely a consequence of ecological pressure in community, and for this reason, de-labelling through either allergy testing or pharmacy-led audit should be encouraged in community and in hospital setting.

## Figures and Tables

**Table 1:** Characteristics of the population.

Characteristics			Overall	*β*-lactam allergy	No *β*-lactam allergy	*p* value
			(*n* = 3332)	(*n* = 132)	(*n* = 3200)	
Biological	Age (years) (mean (SD))		66 (16.4)	64 (15.0)	66 (16.0)	0.15
	Gender (*n* (%))					
		Male	2007 (60.2)	49 (37.1)	1958 (61.2)	<0.01
		Female	1325 (39.8)	83 (62.9)	1242 (38.8)	

Clinical	SAPS-II (mean (SD))		46.0 (20.0)	44.0 (19.1)	46.0 (20.4)	0.21
	Shock (*n* (%))					
		No	1699 (51.0)	69 (52.3)	1630 (50.9)	0.76
		Yes	1633 (49.0)	63 (47.7)	1570 (49.1)	
	Mechanical ventilation (*n* (%))					
		No	1902 (57.1)	79 (59.8)	1823 (57.0)	0.51
		Yes	1430 (42.9)	53 (40.2)	1377 (43.0)	
	Central venous catheter (*n* (%))					
		No	1405 (42.2)	58 (43.9)	1347 (42.1)	0.67
		Yes	1927 (57.8)	74 (56.1)	1853 (57.9)	

Microbiological	MDR strains at admission (*n* (%))					
		Not available	183 (5.5)	3 (2.3)	180 (5.6)	0.04
		No	2762 (82.9)	105 (79.5)	2657 (83.0)	
		One MDR species	361 (10.8)	23 (17.4)	338 (10.6)	
		Two or more MDR species	26 (0.8)	1 (0.8)	25 (0.8)	
	MRSA at admission (*n* (%))					
		Not available	183 (5.5)	3 (2.3)	180 (5.6)	0.21
		No	3034 (91.0)	123 (93.2)	2911 (91.0)	
		Yes	115 (3.5)	6 (4.5)	109 (3.4)	
	ESBL strains at admission (*n* (%))					
		Not available	183 (5.5)	3 (2.3)	180 (5.6)	0.01
		No	2862 (85.9)	110 (83.3)	2752 (86.0)	
		Yes	287 (8.6)	19 (14.4)	268 (8.4)	
	ESBL species at admission (*n* (%))					
		Escherichia coli	196 (68.3)	11 (57.9)	185 (69.0)	0.01
		Klebsiella pneumoniae	45 (15.7)	3 (15.8)	42 (15.7)	
		Other single species	26 (9.0)	5 (26.3)	21 (7.8)	
		Two or more ESBL species	20 (7.0)	0 (0)	20 (7.5)	
	MDR strains at discharge (*n* (%))					
		Not available	187 (5.6)	3 (2.3)	184 (5.8)	0.08
		No	2672 (80.2)	102 (77.3)	2570 (80.3)	
		One MDR species	412 (12.4)	24 (18.2)	388 (12.1)	
		Two or more MDR species	61 (1.8)	3 (2.3)	58 (1.8)	
	MRSA at discharge (*n* (%))					
		Not available	187 (5.6)	3 (2.3)	184 (5.8)	0.18
		No	2996 (89.9)	121 (91.6)	2875 (89.8)	
		Yes	149 (4.5)	8 (6.1)	141 (4.4)	
	ESBL strains at discharge (*n* (%))					
		Not available	187 (5.6)	3 (2.3)	184 (5.8)	0.04
		No	2772 (83.2)	107 (81.1)	2664 (83.3)	
		Yes	373 (11.2)	22 (16.7)	352 (11.0)	
	ESBL species at discharge (*n* (%))					
		Escherichia coli	221 (59.2)	12 (54.6)	210 (59.7)	0.62
		Klebsiella pneumoniae	70 (18.8)	3 (13.6)	67 (19.0)	
		Other single species	52 (13.9)	5 (22.7)	48 (13.6)	
		Two or more ESBL species	29 (7.8)	2 (9.1)	27 (7.7)	
	MDR strains acquisition in ICU (*n* (%))					
		Not available	200 (6.1)	3 (2.3)	197 (6.2)	
		No	3030 (90.9)	124 (93.9)	2906 (90.8)	0.16
		Yes	101 (3.0)	5 (3.8)	96 (3.0)	

Outcomes	MRSA acquisition in ICU (*n* (%))					
		Not available	200 (6.1)	3 (2.3)	197 (6.2)	0.12
		No	3106 (93.1)	127 (96.2)	2979 (93.1)	
		Yes	26 (0.8)	2 (1.5)	24 (0.8)	
	ESBL strains acquisition in ICU (*n* (%))					
		Not available	200 (6.1)	3 (2.3)	197 (6.2)	0.17
		No	3049 (91.4)	125 (94.7)	2924 (91.4)	
		Yes	83 (2.5)	4 (3.0)	79 (2.5)	
	Death in ICU (*n* (%))					
		No	2635 (79.1)	99 (75.0)	2536 (79.3)	0.24
		Yes	697 (20.9)	33 (25.0)	664 (20.8)	
	Days of hospitalization in ICU (mean (SD))		8.1 (9.8)	8.2 (10.0)	8.1 (9.8)	0.87

ESBL: extended spectrum *β*-lactamase; ICU: intensive care unit; MDR: multidrug resistant; MRSA: methicillin resistant *Staphylococcus aureus*; SAPS: simplified acute physiology score II; SD: standard deviation.

**Table 2:** Multivariate analysis for factors associated with allergy to *β*-lactams.

Parameter	RR (95% CI)	*p* value
Female gender	0.4 (0.3-0.6)	<0.001
ESBL strains at admission	1.7 (0.4-10.3)	0.475
ESBL strains at discharge	0.9 (0.2-2.75)	0.573

CI: confidence interval; ESBL: extended spectrum *β*-lactamase; RR: relative risk.

## Data Availability

The data that support the findings of this study are available from the corresponding author upon reasonable request.

## Abbreviations

COVID-19: Coronavirus infectious disease-2019
CVC: Central venous catheter
ESBL: Extended spectrum beta lacamase
ICU: Intensive care unit
MDR: Multidrug resistant
MRSA: Methicillin resistant Staphylococcus aureus
SAPS-II: Simplified acute physiology score II.
